# True Zero-Training Brain-Computer Interfacing – An Online Study

**DOI:** 10.1371/journal.pone.0102504

**Published:** 2014-07-28

**Authors:** Pieter-Jan Kindermans, Martijn Schreuder, Benjamin Schrauwen, Klaus-Robert Müller, Michael Tangermann

**Affiliations:** 1 Electronics and Information Systems (ELIS) Dept., Ghent University, Ghent, Belgium; 2 Machine Learning Laboratory, Technical University of Berlin, Berlin, Germany; 3 Department of Brain and Cognitive Engineering, Korea University, Seoul, Republic of Korea; 4 BrainLinks-BrainTools Excellence Cluster, Computer Science Dept., University of Freiburg, Freiburg, Germany; Centre National de la Recherche Scientifique (CNRS) and Grenoble University, France

## Abstract

Despite several approaches to realize subject-to-subject transfer of pre-trained classifiers, the full performance of a Brain-Computer Interface (BCI) for a novel user can only be reached by presenting the BCI system with data from the novel user. In typical state-of-the-art BCI systems with a supervised classifier, the labeled data is collected during a calibration recording, in which the user is asked to perform a specific task. Based on the known labels of this recording, the BCI's classifier can learn to decode the individual's brain signals. Unfortunately, this calibration recording consumes valuable time. Furthermore, it is unproductive with respect to the final BCI application, e.g. text entry. Therefore, the calibration period must be reduced to a minimum, which is especially important for patients with a limited concentration ability. The main contribution of this manuscript is an online study on unsupervised learning in an auditory event-related potential (ERP) paradigm. Our results demonstrate that the calibration recording can be bypassed by utilizing an unsupervised trained classifier, that is initialized randomly and updated during usage. Initially, the unsupervised classifier tends to make decoding mistakes, as the classifier might not have seen enough data to build a reliable model. Using a constant re-analysis of the previously spelled symbols, these initially misspelled symbols can be rectified posthoc when the classifier has learned to decode the signals. We compare the spelling performance of our unsupervised approach and of the unsupervised posthoc approach to the standard supervised calibration-based dogma for n = 10 healthy users. To assess the learning behavior of our approach, it is unsupervised trained from scratch three times per user. Even with the relatively low SNR of an auditory ERP paradigm, the results show that after a limited number of trials (30 trials), the unsupervised approach performs comparably to a classic supervised model.

## Introduction

In this manuscript, we present our findings from an online evaluation of an unsupervised and calibration-less approach to ERP spelling. For our experiments, we used the basic unsupervised model proposed in [1]. Moreover, in our previous work [1]–[3], this basic model and its extensions were evaluated thoroughly in offline simulations. The promising results in the aforementioned offline studies gave rise to the need for an intensive online evaluation of the unsupervised model, which is the main contribution of the current manuscript. Before detailing the present study, we will take a step back and put our contribution into the appropriate context.

Machine learning (ML) methods capable of extracting information from high-dimensional and noisy data, e.g. the electroencephalogram (EEG), have thoroughly improved the field of Brain-Computer Interfaces (BCI). Before the advent of machine learning, the BCI user was required to complete an intensive training program lasting several sessions [4]. Thanks to the machine learning algorithms this training procedure is significantly reduced [5], [6]. As a result, most healthy BCI users can take control of the BCI (e.g. using a communication application) within a single session.

The contributions of ML methods to the field of BCI are very diverse. For motor imagery tasks and slow cortical potentials, they helped in improving the spatial filtering of electrodes [7], the classification of mental tasks [8], the recognition of error potentials [9] and in solving the feature-/channel selection problem [10], [11]. The recognition of Event Related Potentials (ERP) benefited from the introduction of (regularized) ML methods [12]–[14]. The majority of these methods are so-called supervised methods, and they rely on labeled data to train the algorithm. Hence, calibration session, during which the user is instructed to perform specific tasks (e.g. focusing on a specific stimulus or imagining a movement of the left hand), is required to obtain these labeled datasets.

Due to the dependence on these time-consuming calibration recordings, state-of-the-art BCI systems have difficulties coping with the limited attention span of some patients in need of a BCI [15]. This problem is well recognized by the BCI community, as evidenced by the multitude of mitigation strategies for both self-driven paradigms e.g. motor imagery tasks, and paradigms relying on attention-modulated ERPs that are elicited by external stimuli. Common strategies comprise: sharing classifiers between users [16]–[22] or between sessions of the same user [22], [23], the utilization of more salient stimuli [24]–[28] and improved experimental paradigms [29]–[31]. Overall these methods aim to avoid or at least shorten the required calibration time. Additionally, approaches aiming to increase the speed at which the user interacts with the BCI have been proposed. Examples include dynamic stopping procedures for ERP paradigms [32], [33] and the use of shared control of for example a robotic wheelchair [34]. Other improvements involve the incorporation of elaborate language models for communication applications [35]–[37].

When combined, the aforementioned approaches alleviate the problematic situation but they are not always sufficient – for example, when the labeled calibration data itself is an outlier measurement or when there is a different type of non-stationarity in the data (e.g. due to fatigue). In this case, the knowledge obtained on the calibration data by the ML model does not allow for reliable decoding of the *normal* data in the following online runs. To compensate for this type of non-stationarity, researchers have proposed online adaptation strategies [22], [23], [38]–[41]. Many types of ML models rely on valid estimates of the covariance matrices of the data and of a bias term. As labels are not available during the online use of the BCI, several of these strategies are limited to the adaptation of the bias and the *pooled* covariance estimate instead of the desired *class-wise* covariance matrices. Furthermore, these approaches still require a calibration session since they are based on supervised trained classifiers, e.g. linear discriminant analysis (LDA) or support vector machines (SVM).

In the current manuscript, we go beyond these basic adaptation strategies and argue in favor of a completely unsupervised classification approach recently proposed by Kindermans et al. [1]. The unsupervised classifier starts out randomly and learns online from unlabeled data. As a result, it abandons the need for a time-consuming calibration recording. Furthermore, the adaptive nature of the classifier allows it to adjust to changes in the recorded data. In contrast to the common adaptive approaches [16], [17], [22], [41], the method analyzed here does not depend on pre-trained classifiers or data sets from previous sessions. Nevertheless, the classifier can make use of such data if available, as shown in offline experiments [2], [3].

In previous work, the unsupervised model and its extensions have been evaluated extensively offline on visual ERP data [1]–[3]. The promising results obtained in these offline studies elicited the need for a thorough online evaluation of the unsupervised approach. The importance of an online evaluation is threefold. First, the true test of the reliability and robustness of a machine learning based decoder for BCI is an online evaluation. Offline analysis can only provide an estimate of the performance. Therefore, before moving on to patient studies, one has to ascertain whether the proposed model performs reliably in online experiments. Second, it demonstrates that it is indeed possible to integrate the unsupervised decoder in a online BCI setup. Finally, an offline study can only investigate the adaptation of the machine learning algorithm to the user. Only an online study enables us to verify whether the two-way man machine interaction and adaptation during BCI usage is successful.

This manuscript investigates in how far the established supervised classification dogma (including time-consuming calibration recordings) can be replaced by the unsupervised approach. On top of that, instead of a visual ERP paradigm, for which Kindermans et al. have shown good offline results, we make use of an auditory ERP paradigm AMUSE proposed by Schreuder et al. [42]. This auditory ERP paradigm increases the difficulty of unsupervised learning because its auditory evoked potentials (AEP) have a lower SNR than those of visual paradigms [43]. We evaluate the unsupervised method on an online copy spelling task, which is performed by ten healthy BCI users with normal hearing. To assess the learning behavior of the unsupervised classifier, it is being re-set to random parameters three times during the course of the experiment.

## Methods

### Ethics statement

Despite not being a medical research study, it involved human subjects. Thus we followed the ethical principles of the WMA Declaration of Helsinki. Approval was requested for and granted by the local ethics committee of the Charité Universitätsmedizin, Berlin, Germany. Participants were compensated with 8 EUR/h for participation. They received detailed written information about the experiment days in advance. Before the start of the recording session, participants declared written informed consent for participation and the use of their data in anonymized form. All recorded data was anonymized during the registration.

### Experimental setup

Our experimental setup was designed to compare a classic supervised calibration based approach to a calibration-less model based on unsupervised learning. Please note that the supervised method requires labeled data, hence the requirement for a calibration session. The unsupervised model on the other hand does not require labeled data, as a result it can be used without calibration. Obviously, unsupervised learning is not the only approach to building a calibration-less BCI. Indeed, cross-subject transfer learning, where classifiers are pre-trained on a set of different subjects and subsequently applied to a novel subject (e.g. [16]–[22]), is also a valid and increasingly more studied option. Nevertheless, since this manuscript considers the evaluation of a specific unsupervised calibration-less approach and a supervised calibration based approach, we will use the specific machine learning terms unsupervised and supervised throughout the remainder of the manuscript.

### AMUSE

Up to a few small changes, the present study followed the spatial auditory ERP paradigm AMUSE proposed in [42], that uses six different tones as stimuli. In this paradigm, the participant is surrounded by six speakers, one for each tone. These six stimuli/tones (40 ms duration) can be uniquely identified by making use of two sources of information: its unique pitch and its unique presentation direction (for more details on this double-cueing paradigm, we refer the reader to [42]). Compared to the original AMUSE publication, two changes to the ring of speakers are implemented to reduce the probability of front-back confusions between stimuli (cone of confusion). First, the position of the loudspeakers was modified, in this study they are not equally spaced on the ring, but they are slightly shifted towards the front. Second, the participant is placed about 10 cm behind the center of the ring and not in the center itself.

The AMUSE publications [42], [44], [45] have shown that a BCI with a purely auditory interface is possible. As the focus of the present study was on the comparison of a supervised calibration-based method and an unsupervised calibration-less data processing approach (see below), the spelling interface had been simplified and is supported by information displayed on a screen in front of the participants. But during the stimulus presentation, the information on the screen was static.

**Figure 1:**
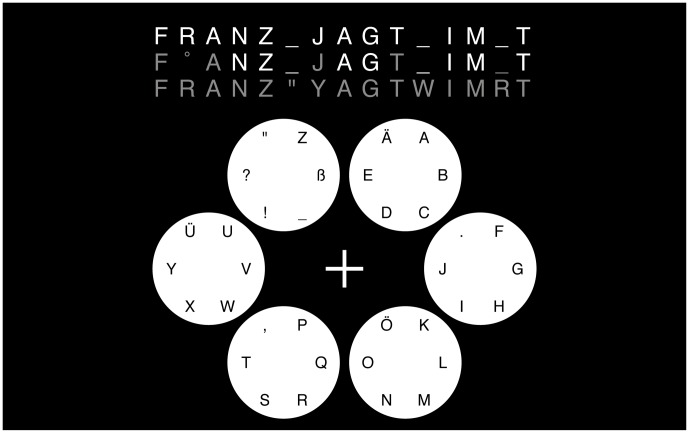
Drawing of the user interface at the end of a unsupervised block of 15 letters (30 trials). The target text in the first line is always present for the subject. Text spelled with the unsupervised method appears letter-by-letter in the second line, and the text after re-analysis with the posthoc method is shown in the third line. In the lower part of the screen, circle positions visualize the auditory scene from a top view. Each circle encodes one out of six tones/tone directions relative to the user, who is positioned in the middle of the ring of speakers (fixation cross). After selecting a group of letters (duration: one trial), the user virtually moves into the corresponding circle and can select a letter from within this circle by a second trial.) The result shown in this figure corresponds to the first unsupervised block of subject *nbf*.

By using the six tones/directions, the BCI allows to make a one-out-of-six selection at the end of each trial. A two-step selection procedure allows for spelling one of 36 symbols. These include 26 letters, German umlauts, punctuation marks as well as an underscore symbol that represent white space. Writing a symbol was performed by selecting one out of six groups of symbols in the first step, and one of the six within-group symbols in the second selection step. The exact grouping of the symbols is shown in Fig. 1.

#### Data acquisition

The EEG was recorded and stored at 1 kHz from 31 passive Ag/AgCl electrodes against nose reference using BrainProducts BrainAmp amplifiers. Following the extended 10–20 naming scheme, these were channels Fp2, F9, F5, F1, F2, F6, F10, FT7, FC3, FCz, FC4, FT8, C5, C1, Cz, C2, C6, TP7, CP3, CPz, CP4, TP8, P9, P5, P1, P2, P6, P10, POz, O1 and O2. The built-in filters performed a band-pass with a 0.1 Hz lower bound and 250 Hz for the upper. Any further processing of the data was preceded by additional low-pass filtering at 45 Hz, and down-sampling to 100 Hz.

Recording one additional EOG electrode below the right eye, it became possible to calculate the bipolar vertical EOG using the EEG channel Fp2, and a horizontal bipolar EOG using EEG channels F9 and F10. For any of the following signal processing and classification steps, only the 31 EEG channels (including Fp2, F9 and F10) were used, not the single vertical EOG channel nor any bipolar EOG channel.

#### Participants

Ten healthy, external participants (four females, six males) with an average age of 34.2 years (median: 30.5, min: 20, max: 58) were recruited by an online advertisement to avoid a bias towards university students. These subjects are represented by the codes *nbb, nbc, nbd, nbe, nbf, nbg, nbh, nbi, nbj, jh*. They stated to have normal hearing, to be non-smokers, to have no known neurological disorder or history thereof, and that they do not take any psychoactive or EEG-altering substances. However, a systematic test thereof was not performed. The individual background of musical education (playing an instrument or singing) varied strongly and ranged from 0 to 36 years (time accumulated over instruments). Eight subjects had no prior experience with BCI or EEG experiments. Participant *nbj* had participated in an earlier EEG experiment at a different research lab, but this was not BCI-related. Participant *jh* participated in an earlier motor-imagery BCI study of our lab (i.e. the Berlin BCI group). Every participant received information about the course of a session about one week in advance. This included task instructions, the request to have a good night's rest the night before, and a morning hair wash on the day of the experiment. Except for one unreported participant in a pilot recording of a reduced version of this experiment, no other subjects participated in the study.

#### Course of the experiment

**Figure 2:**
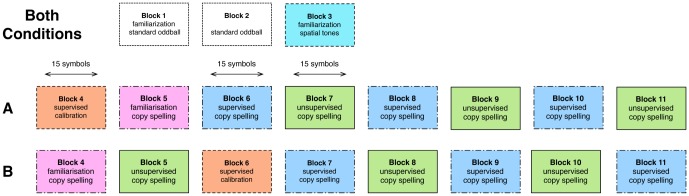
Schematic display of the course of an experimental session.

Each participant performed a single session of approximately 4 hours. This included informing the participant, receiving consent, the setup of the EEG cap, detailed instructions, the actual recordings, the wrap-up and a hair wash. The course of the experimental session itself is visualized in Fig. 2. In the first block, we educated the participant about the standard auditory oddball recording. In the second block, we performed two auditory oddball recordings. In the third block, we familiarized the subjects with the concept of the spatial tones. After these three introductory blocks, participants were assigned alternately into one of the groups A and B.

Members of group A performed a calibration session for the supervised classifier in the fourth block. In the fifth block, they were instructed on how to use the spelling interface itself. This allowed them to perform the copy spelling task in the final six blocks, alternating between evaluation of the supervised model and evaluation of the unsupervised model.

Members of group B got immediately acquainted with the spelling interface. The fifth block was the first evaluation run of the unsupervised model. The sixth block contained the calibration session for the supervised model and was followed by the first evaluation of the supervised model in the seventh block. To complete the session, the members of group B performed four more evaluation blocks switching between the unsupervised and supervised methods.

What follows is a more detailed description of the experimental blocks.

#### Standard oddball familiarization and recording

After the cap setup, the users were shown their ongoing EEG signals and were taught how to avoid typical artifacts, e.g. as eye-blinks, during the recordings. In the following, all of the users were first introduced to, and then performed two standard auditory oddball recording. Each odball recording lasted five minutes. It comprised a stream of two short tones originating from a single speaker (from a front-right direction) with 1000 ms stimulus onset asynchrony (SOA) and a 1 to 4 ratio between the high-pitched target and low-pitched non-target tones. The participants were asked to count the number of target tones (40 per block) silently and motionless.

#### Familiarization with the spatial tones

After the standard oddball recording, participants were familiarized with the spatial auditory setup of the AMUSE paradigm. The participants heard examples of tone sequences presented from the ring of six loudspeakers around them. The stimulus presentation started with a slow SOA of 1000 ms. This was subsequently reduced to a SOA of 175 ms, which equals the SOA used during the real experiment.

#### Classifier calibration

During the calibration block, participants were repeatedly asked to focus their attention on one of the six tones/directions. The target tone was indicated prior to trial start by three tone cues and supported by a visualization of the target direction on the computer screen in front of the participant. After a short break of 2 s, a rapid stimulus sequence was presented. It consisted of 15 iterations of six tones, resulting in 90 tones. The tones had a duration of 40 ms and were presented in pseudo-randomized order with a SOA of 175 ms. During the stimulation sequence of 15.75 s, the participant counted (internally and motionlessly) the number of target tone appearances (15) while they tried to neglect non-target tones (75).

#### Familiarization with copy spelling

Before the first copy-spelling block, participants were familiarized with the copy-spelling application that had to be controlled using spatial auditory attention. By using six tones/directions, the BCI allows to make a one-out-of-six selection at the end of each trial. To spell one of the 36 symbols, they required two trials. In the first trial, they were able to select a group of symbols. In the second trial they were able to select a symbol from this group. The set of possible symbols consisted of 26 letters, German umlauts, punctuation marks and white space (represented by an underscore).

#### (Un)supervised copy spelling

After familiarization with the interface, the real copy-spelling runs began. During the online spelling phase, one out of two classification methods was applied online to decode the spatial attention and thus determined the copy-spelled symbols. The decoding method alternated between the supervised and unsupervised methods during the six online writing blocks. This resulted in three pairs of two blocks, where within each pair each method was used once.

During each of the six online evaluation blocks, the participants were asked to copy-spell a string of 15 symbols (split into three sub-blocks of 5 symbols) by performing 30 selection trials. For a better comparison between the two methods, the same target text was used during the two blocks of a pair. The text was pre-defined for the first two pairs (FRANZ_JAGT_IM_T and AXI_QUER_DURCH_), and was chosen freely by the user for the last block pair.

The user interface for the copy spelling task is shown in Fig. 1. The target string is show on the top of the screen (first line). The participant had to try to re-produce it symbol by symbol. Wrong selections could not be undone, as the spelling interface did not allow for *undo* actions – neither on the group level nor on the letter level. The copy-spelled symbols appeared one by one in the second line as the trials were completed. This output was fixed, it did not change after the initial prediction. Depending on the decoding success of the BCI system, a symbol in the second line was either displayed in white (providing that both selection steps were correct) or in gray otherwise. A special character (°) displayed in gray indicates that both selections had failed. An additional third line of symbols in gray was displayed during blocks of the unsupervised method. This line contained posthoc re-estimates of the string spelled so far. This complete string of this third line was updated after each trial. Participants were instructed about the first two lines and told to ignore the changing third line.

### Data Preprocessing

Online data preprocessing was nearly identical for both classification approaches. The 31-channel EEG data was low-pass filtered causally to below 40 Hz by a Chebyshev type 2 filter of order five (stop-band attenuation of 20 dB), and an epoch from -200 ms to 700 ms relative to the stimulus onset was extracted for each tone stimulus. The baseline activity of each channel and stimulus epoch was estimated from the pre-stimulus interval of [−200 ms 0 ms] and subtracted. For the data of the calibration block only, outlier epochs were removed based on a variance criterion, while during online use all epochs were kept. The variance criterion rejects all epochs where the variance within that epoch is higher than 2.5 times the variance threshold. In our case, the threshold is equal to the 90th percentile of the variance of the epochs.

For classification, twelve features were extracted from each channel, the intervals (in ms) were [100 130], [130 160], [160 190], [190 220], [220 250] for earlier, more transient ERP components, and [250 300], [300 350], [350 400], [400 450], [450 500], [500 600], [600 700] for later, slower components. After concatenation, they formed a 372-dimensional feature vector for each epoch. The features of a channel consisted of the average potentials of these twelve time intervals post stimulus. The interval borders were chosen to generally capture the class-discriminative ERP information.

For the unsupervised classification method two minor additional steps had to be included for technical reasons. First, normalization to zero mean and unit variance was applied feature-wise per trial. Second, the inclusion of a bias term was necessary. This bias term is a constant feature equal to 1. Including it allows us to control the offset of the classifier directly.

Offline data processing for supervised training and for the visualization of the grand average physiology included the removal of outlier epochs and outlier channels based on a variance criterion. Per subject, 100–300 epochs and 0–2 channels were removed. Furthermore, an acausal forward-backward bandpass filter was applied (0.5–20 Hz) and baseline activity was removed prior to computing average ERP responses. Class-discriminant information is displayed using signed and scaled *area under the ROC curve* values (ssAUC), such that ssAUC = 0 corresponds to AUC = 0.5 and most extreme AUC values are mapped linearly to ssAUC values of −1 and 1: 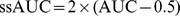.

### State-of-the-art: supervised classification

To represent the state-of-the art of a calibration-based, supervised trained classifier, a subject-specific linear discriminant analysis (LDA) classifier was used in combination with shrinkage-regularization on the sample covariance matrices [46]. This type of classifier is known to perform well on a wide range of ERP-BCI paradigms including those with visual, auditory and tactile stimuli [37], [47]–[49]. It was trained on the data of 30 labelled trials collected during one block of calibration. Please note that thirty trials correspond to 2250 target epochs and 450 non-target epochs, from which about 10% had been removed on average by the above mentioned outlier removal preprocessing. The resulting classifier was used during three spelling blocks. It was not adapted during its online use and thus could potentially suffer from non-stationarities over the course of the experimental session.

### Unsupervised classification

To allow for unsupervised learning in ERP spellers, we embed prior knowledge about the ERP paradigm directly into the probabilistic model [1]. We explicitly model the fact that the user has to focus on a single stimulus during an entire trial. It can be assumed that this and only this specific stimulus (and all repetitions thereof) will result in a target ERP response, and that any other stimulus presentation evokes a non-target response. This assumption reduces the problem difficulty and enables us to perform unsupervised learning by performing inference at the level of the desired stimulus and not in a binary target vs. non-target setting. This becomes clear when we consider the number of possible solutions for the labeling problem. Given 6 different stimuli and 15 iterations per trial (90 stimuli in total), there are just 6 ways to select the desired stimulus. But there are 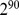 possible labellings of the stimuli in a binary target vs non-target setting. On top of that, limiting the solutions to those that are feasible according to the paradigm, guarantees us to assign the right label to either all or 4 out of 6 stimuli. When we make a mistake, then we have swapped the label for the target and a non-target stimulus. The label for the four remaining non-target stimuli is still assigned correctly. In this model, we assume that each stimulus has equal probability of being the desired one, but this can be extended easily to include prior information from language statistics [2], [3], [35]. Additionally, the model assumes that the 1-D projection of the ERP features is Gaussian with a class-dependent mean and shared variance. This is slightly more general than the assumption made by LDA, where the data is assumed to be Gaussian in the original high-dimensional feature space. Furthermore, the 1-D projection of a multivariate Gaussian is always Gaussian, but the 1-D projection of non-Gaussian distributed variables can be Gaussian too. The distribution of the one-dimensional projection of the EEG features will be used as an approximation of the distribution of the EEG itself. This approximation reduces the computational complexity of inference and classifier updates. Finally, we add regularization by placing a zero mean, isotropic covariance prior on the classifier's weight vector. This prior restricts the classifier to simple solutions by keeping the weights small.

#### Defining the probabilistic model

Next, we introduce the notation. The attended stimulus during trial 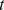 is 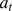. There are 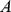 different stimuli, where 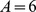 in the case of the AMUSE paradigm. During stimulus presentation 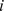 for trial 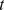 stimulus 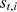 is presented to the user. The function 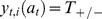 encodes that when the attended stimulus is presented, it has to be a target stimulus, if a different stimulus is presented then it must be a non-target stimulus. Let 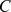 be the number of channels and 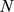 the number of samples used per channel, then the 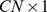 dimensional EEG feature vector for stimulus 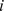 during trial 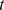 is denoted by 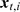. This vector 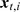 is projected towards 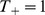 when it is associated with a target stimulus, and towards 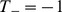 if it is a non-target. The distribution of the projected EEG features has mean 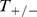, depending on target or non-target and precision 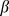. The 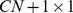 dimensional weight vector used to project the EEG is 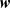. Note that the additional term corresponds to the bias. The prior distribution on this weight vector has zero mean and precision 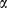. Furthermore, let 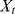 be the matrix containing all the feature vectors for trial 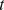, one feature vector per column. Let 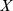 be the matrix containing all the feature vectors recorded up till this point. Finally, 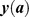 is the vector containing the target vs. non-target encoding for all the feature vectors in 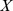.

Using the notation from above, the model is defined as follows.
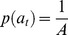





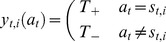





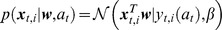





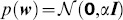
The term 
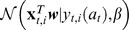
 denotes a normal distribution with mean 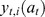 and precision 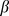.

#### Inferring the desired symbol

When we have a trained model, we can infer the probability that a specific stimulus is being attended by applying Bayes's rule:
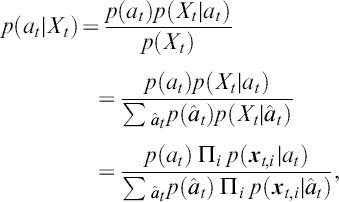
where we predict the stimulus with the highest likelihood.

#### Unsupervised training

We use the Expectation Maximization (EM) algorithm [50] to optimise 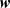 and 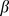. The attended stimuli are unknown and have to be inferred during the expectation step. Optimizing 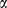 is easier as it depends only on 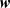, thus direct maximum likelihood can be used. The resulting optimization process uses the following update equations.
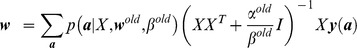





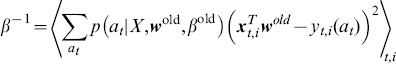





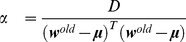
The update equation for 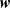 can be seen as a weighted sum of all possible ridge regression classifiers, weighted by the probability that the labels used to train the classifier are correct given the previous estimate of 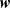. The update for 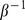 is the expected mean squared error between the projected feature vectors and the target feature vectors. Thus, 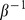 equals the expected variance of the projected feature vectors. Finally, the precision 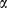 is set to the inverse of the average squared classifier weight. Furthermore, we would like to stress that even though we train the classifier to detect the attended stimulus directly, a classifier which discriminates between target and non-target responses is embedded into the model.

#### Practical usage

There is one big caveat when training a classifier without label information. It is impossible to control what the classifier actually learns, as the underlying algorithm tries to maximize the likelihood of the data under the current model. Therefore, it is possible that the classifier learns to solve the exact opposite problem, i.e. it swaps the target and the non target labels for the individual stimuli. However, it has been shown that there is a strong correlation between the data-log likelihood and the selection accuracy or the AUC [1]. To counter this problem, we adopted the following approach, which had originally been proposed in [1], during the online experiments: We initialize five different classifier pairs. For each pair, we draw 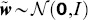 and we initialize one classifier with 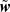 and one with 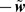. Hence one classifier per pair can be expected to perform above chance level and one classifier will be below chance level in terms of AUC for labeling the individual feature vectors. After each trial, we perform five EM iterations per classifier. Due to the initialization, we expect that on average at least one classifier will learn to solve the desired task and one classifier will learn the opposite task. Subsequently, we select the best classifier with respect to the data-log likelihood to predict the attended stimulus. After predicting the attended stimulus, we update the classifier pairs. Per pair, we select the classifier with the highest data-log likelihood. Let 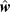 be its weight vector. Then we re-initialize the other classifier of the pair with 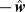. This ensures that one classifier per pair will perform above chance level and one will perform below chance level. Using this strategy, we maximize the chance that at least one classifier would solve the task correctly. For correctness and reproducibility, we would like to mention that there was a minor mistake in the implementation of the log likelihood. We used log_lik  = −0.5*log(sqrt(2*pi*sigma))−0.5*((X-mu).^2)/sigma; instead of log_lik  =  -log(sqrt(2*pi*sigma))−0.5*((X-mu). ^2)/sigma;. We verified trough offline simulations that it did had not affected the experimental results. Furthermore, there are different options to select the best classifier, e.g. selecting the classifier where the expected mean squared error between the target label and the actual projection is minimal is also possible.

### Unsupervised posthoc classification

When the classifier is used during an online experiment, it accumulates more and more unlabeled data to train on. As a consequence, the quality of the decoding model improves as more trials have been processed. Hence, a re-analysis of the stimuli of the previous trials may lead to different outcomes compared to the original (online) predictions. This so-called *posthoc* re-analysis of preceding trials can be done easily during the online experiment. Re-evaluating all previous trials (in addition to the current trial) allows us to measure accurately how successfully the classifier has adapted to the user.

Furthermore, this posthoc analysis strategy can provide an additional benefit to the user during the spelling task by accepting that the unsupervised classifier might initially make mistakes on some of the letters. These faulty decisions of the initial classifier might be revised during the subsequent posthoc re-analysis. The user can expect the posthoc classifier to correct the initial mistakes in the output during the course of the online experiment. Consequently, a user would require a <BACKSPACE> or <DELETE> functionality of the spelling application only when he made an error himself or when he changed his mind about the already written text.

### Hyperparameters

The data from the original AMUSE study [42], which comprises 21 subjects, was used in an offline analysis to determine the hyperparameters of the classification methods. The pre-analysis showed, that the methods performed stable, and with good results for a large range of values.

For the unsupervised method, we opted for 5 classifier pairs, and the number of EM updates per trial was fixed to 5 as well. Additionally, we chose to initialize 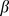 to 1 and 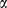 to 100. During the experiments, the value of the regularization parameter 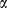 was limited to at most 200 to prevent the classifier from collapsing on the degenerate solution of a vector of zeros. This is a practice that was suggested in the original paper [1].

The number of stimulus iterations per trial was not determined in a data driven approach. It was set to 15 to match the original AMUSE study. The number of trials for both the calibration block and the online blocks was 30. This value was selected based on our prior experience with the supervised method. More than 30 trials of calibration data would not lead to a significant further improvement of the classifier on the grand average of the original AMUSE data.

## Results

### Basic Neurophysiology

**Figure 3:**
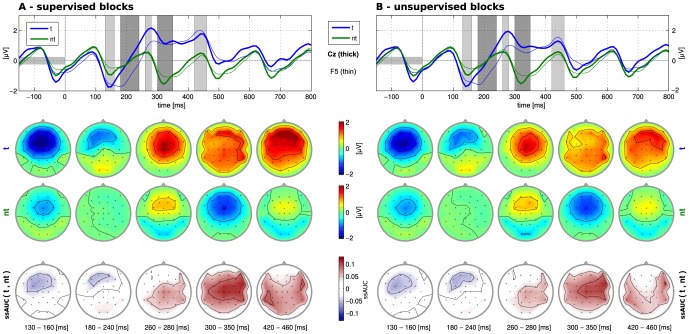
Grand average ERP responses (n = 10) for supervised (left) and unsupervised online spelling blocks (right). Top row: Responses evoked by target (blue) and non-target (green) stimuli for channels Cz (thick) and F5 (thin). Middle row: Scalp plots visualizing the mean target (t) and non-target (nt) responses within five selected time intervals (see grey markings of the top row from 130 ms to 460 ms post stimulus). Bottom row: Scalp plots visualizing the spatial distribution of class-discriminant information, expressed as the signed and scaled area under the receiver-operator characteristic curve (ssAUC).

For both the unsupervised and the supervised recordings, very similar ERP responses are observed in the grand average analysis (left and right plots of Fig. 3). This is a first indicator that the online performance differences between the methods (see below) are not caused by differences in the recorded data. In both conditions, typical attention-related and class-discriminative differences can be discerned: a fronto-central negativity between 100 and 200 ms post stimulus and a positivity from 250 ms onwards. Compared to the original AMUSE setup, the target- and non-target ERP-responses of fast auditory ERP paradigms were reproduced in the current study — despite the minor changes in the experimental setup.

### Online performance and block-level temporal dynamics

**Figure 4:**
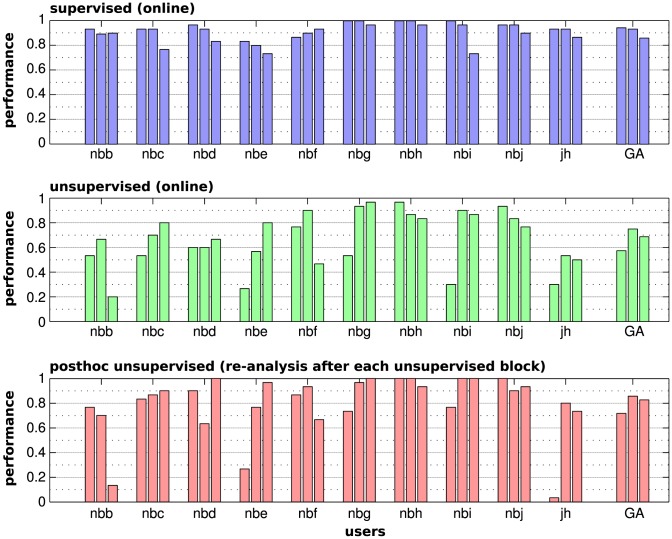
Performance comparisons (trial-based selection accuracy). For each user and the grand average (GA), the performances of three experimental blocks are given. Chance level performance is at 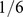. **Top plot**: Online performance of the three blocks per user classified by the supervised LDA approach. Per subject, the classifier had been pre-trained on calibration data (not shown) and kept fix for all three blocks. **Middle plot**: Online performance of blocks controlled by the unsupervised classifier. The unsupervised classifier had been initialized randomly before each individual block (three times per subject). **Bottom plot**: performance of the posthoc re-analysis method for the unsupervised blocks. The posthoc classifier, too, had been initialized randomly before each block.

In our experiments, two (flawless) trials are needed to spell a symbol (correctly). We begin by presenting the trial-wise selection accuracies from the online experiment in Fig. 4. For each subject and each condition, the accuracy is given per block.

#### Supervised

Averaged over all experimental runs, which comprises 30 experimental blocks (10 users times three blocks) with 30 trials per block, the pre-calibrated baseline method supervised obtains a selection accuracy of 92.1% (see the top row in Fig. 4). The minimum accuracy of a block is 73% and it is at least 80% in 27 out of 30 blocks, at least 90% in 21 blocks and all trials are decoded without flaws in five blocks. Due to the fixed classifier, the performance is relatively stable over the three supervised blocks. Increased fatigue had been reported by a number of participants for the last blocks. This may have lead to the slight performance drop from the second to the third (last) block. Despite its small average difference, it was found to be significant with a paired t-test t(9) = 2.91, p = 0.02.

Based on the supervised classifier alone, it can not be explained how fatigue might have influenced the classification performance. We present two hypotheses: first, the changed mental state lead to non-stationarity in the EEG, but the actual attention task is still performed well by the fatigued users. As a consequence, the fixed classifier has more difficulty to decode the trials of the last block, as the non-stationarity in the EEG disturbs the decoding e.g. via changes in the covariance structure of the data or changes in the background EEG. Second, the class-discriminative information contained in the last block might be reduced due to attention deficits of the users, which would result in a reduced SNR due to a less informative *signal* component. We will show later, by simulating an extended experimental session with the unsupervised method, that the SNR is not reduced and that the data can be decoded reliably.

#### Unsupervised

In the short online blocks, the randomly initialized unsupervised method did not reach the performance level of the supervised classifier. However, with 67% accuracy on average, it is far above chance level (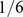). Furthermore, its selection accuracy was at least 70% in half of the 30 unsupervised blocks and 80% or more in 12 of them. The best six unsupervised blocks were completed with an online selection accuracy of no less than 90%. But there is a large amount of variability between the different users. The best result was obtained during the first unsupervised block of user 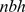, where only the second out of 30 trials was faulty. User 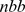 on the other hand obtained the worst result during his final unsupervised block where only 20% of the trials are decoded accurately. Similar to the supervised method, the third (last) unsupervised block has on average a decreased selection accuracy compared to the middle block, but contrary to the supervised case, this is not statistically significant t(9) = 0.90, p = 0.39. In addition, the average unsupervised performance is increased from the first to the second block. An effect that was statistically significant t(9) = −2.54, p = 0.03, but had not been observed for the supervised method.

On the individual level, a substantial amount of variability between unsupervised blocks of the same user is observed. Participant 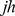 for example was not able to gain control in the first unsupervised block but achieved 80% selection accuracy in the second unsupervised block.

### Online text entry

Under the hard testing conditions (re-initializing the unsupervised classifier to random values before the start of each block), the unsupervised classifier performs at chance level at the beginning of each block. The performance improves dramatically towards the end of a block, resulting in 7.80 out of 15 (52%) correctly spelled symbols for an average unsupervised block. In the posthoc condition, the classifier performance increases to an average of 10.37 out of 15 symbols (69%) per block. This improvement is possible, as the posthoc classifier has had the possibility to learn from data of the full block (15 letters/30 trials) before estimating the written symbols. As a comparison, the pre-trained supervised classifier manages to spell 12.88 out of 15 symbols (86%) without error.

**Figure 5:**
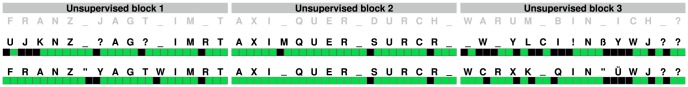
Spelling results for subject *nbf* during the unsupervised blocks. Per block, the top line represents the desired text, the middle line displays text produced online by the unsupervised classification. Text predicted by the posthoc re-analysis at the end of the block is shown at the bottom line. Two trials are needed to determine a symbol. Individual selection errors (wrong trials) of both methods are marked by black squares directly below each symbol. Please note that the classifier was re-initialized randomly at the beginning of each block.

To give the reader a feeling of the spelling quality of the unsupervised approach, Fig. 5 presents the texts spelled (in German) by an average-performing subject *nbf* during the three unsupervised blocks. It is clear that even with a selection accuracy of nearly 80% in the first block, it is difficult for a human observer to make sense of the spelled text. A selection accuracy of around 90% in the second block results in better readability. In the first two blocks, the posthoc classifier was able to revise a substantial number of symbols which had been predicted erroneously during the course of the experiment. Even though it introduced new errors, the amount of wrongly decoded symbols is reduced by 40%, from ten to six.

Unsupervised learning is a significantly more difficult problem than solving the decoding task with a supervised classifier. This was amplified by re-initializing the unsupervised classifiers randomly at the beginning of each block. As a result, some blocks could not be decoded properly by the unsupervised methods, while the supervised classifier succeeded to do so. As an example, the performance was rather poor in the third unsupervised block of subject *nbf* and even the posthoc re-analysis was not able to correct the output to a human-readable level within these 30 blocks. When we applied the supervised classifier to this block (in an offline analysis), it was able to perform well on this block. If, however, the information content is rather high, then the data of 15 trials is sufficient to obtain a good solution unsupervised. The second block of subject *nbf* can be taken as an example — here the selection accuracy of the posthoc re-analysis was equal to the best supervised result for *nbf*.

To judge the value of the three methods we should not be restricted to the spelling accuracy on short blocks. The invested amount of time is an important factor, especially for patient applications. At the moment of the posthoc re-analysis (e.g. at the end of an unsupervised block), a user has spend the same amount of time interacting with the BCI as if he would have performed one full calibration run. While the calibration recording cannot result in any usable text output, the unsupervised block can. On average, it allows a user to communicate straight away with 2/3 of the symbols decoded correctly. We are aware, that this rate is not yet enough to communicate in practical situations. On the other hand, the remedy is simple: as we will show later on in a simulated time-extended experiment, most of the errors can be sorted out by posthoc if the spelling duration is prolonged.

### Within-block warm-up dynamics

**Figure 6:**
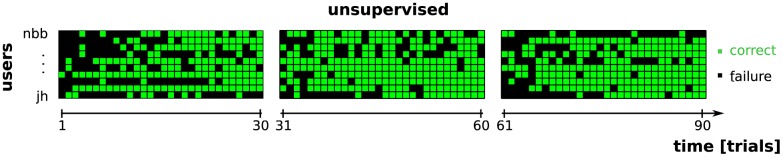
Evolution of errors performed over time (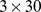 trials) by the unsupervised method for the three unsupervised blocks. Time is on the horizontal axis, while the lines represent users. The order of the users equals that of Fig. 4, with 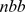 represented by the top line and 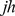 by the bottom line. For each trial and user, a green square indicates an accurate selection, a black one marks an error. Clearly, the unsupervised classifier commits most erroneous decisions shortly after its random initialization at the beginning of each novel block. In the majority of cases users were able to effectively control the BCI by the end of a block.

Now we return to the trial-based performance, and analyze the dynamic behavior within each of the 30 online blocks. The unsupervised method undergoes a constant learning process during the online usage of the BCI. It reveals a so-called warm-up period even on a single-subject basis (Fig. 6). This period explains the reduced performance compared to supervised: unsupervised makes more mistakes during the beginning of each block than at the end.

Hence, it is an important question, how long an average user takes to obtain control over the BCI with the unsupervised approach. We define that a user is able to control the BCI as soon as three consecutive selections/trials are decoded without mistake. The probability to do so by guessing is only 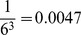. The exact point in time where the user takes control of the BCI for the first time is defined as the first trial of the first sequence of 3 error-free trials. By applying this definition, only three runs ended without a user reaching control. These were the runs of 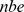 and 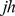 in the first unsupervised block and the run of 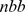 during the third unsupervised block. For the other runs, the average number of trials necessary to achieve control was 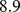. Two runs resulted in control in the very first trial. Furthermore, in 50%, 70% and 90% of all runs, the users were able to control the BCI within 5, 15 and 25 trials respectively.

**Figure 7:**
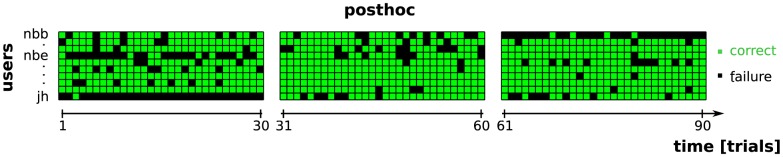
Selection errors committed by the posthoc evaluation method after having processing the data of one entire block. The data displayed stems from the same blocks as in Fig.6, which had been recorded while feedback was given by the unsupervised method. With the exception of three difficult blocks (first blocks of users *nbe* and *jh*, and third block of user *nbb*) the posthoc re-analysis obviously outperforms the original online performance gained by the unsupervised method (see Fig.6). It effectively corrected most initial mistakes at the beginning of each block, thus recovering communication from the very first trial on. Both unsupervised methods (online and posthoc) had trouble selecting a good performing classifier for the three difficult blocks.

As we discussed before, the post-hoc re-analysis re-applies the final classifier to all trials after processing the entire block. In the actual experiment, post-hoc was also used to compute an updated estimate after each trial. The final updated prediction achieved an average selection accuracy of 80%. In 25 out of 30 blocks it obtained a selection accuracy of at least 70%. An accuracy above 80% was reported in 19 blocks and in 15 blocks post-hoc predicts the attended stimulus in at least 90% of the trials. Finally, during seven out of 30 blocks, the post-hoc method was able to present and error-free decoding of the entire block. The block-wise selection accuracy for post-hoc is shown in Fig. 4 and the individual errors are visualized in Fig. 7, where we see that post-hoc was able to correct most of the mistakes made during the online experiment. Unfortunately, for the three blocks that did not result in control in the unsupervised setting, the post-hoc re-analysis failed too.

For two of these three cases, we received specific comments by the users. User *nbe* reported after the first online block that during this block, which happened to be with the unsupervised classifier, she had trouble ignoring one very salient tone (front-left). In the questionnaire at the end of the session she reported, that this problem did not persist during following blocks as she had found an internal strategy to concentrate better on target tones. User *jh* reported, that during his first online spelling block (which happened to be with the unsupervised classifier) he had trouble ignoring one very salient tone (front-right). However, he got used to it or found a different mental strategy and reported that the problem was solved in the following blocks. Nothing was reported by user *nbb* which could explain the performance break-down in the third unsupervised block (which happened to be the last block overall for this user).

In the next section we will demonstrate by means of a simulated experiment of an extended online spelling session that even these blocks contain enough information to allow for reliable decoding of the EEG — with both the unsupervised and the supervised methods. Hence, the decoding problem for the unsupervised approach is not caused by non-informative EEG signals, but rather by the combination of a relatively short block duration and a rather low signal to noise ratio. This combination prolongs the warm-up period of the unsupervised classifiers. In general, the less data available the harder it is to learn without label information, and this is amplified when the data has a low signal to noise ratio.

### Simulated online experiment of extended duration

**Figure 8:**
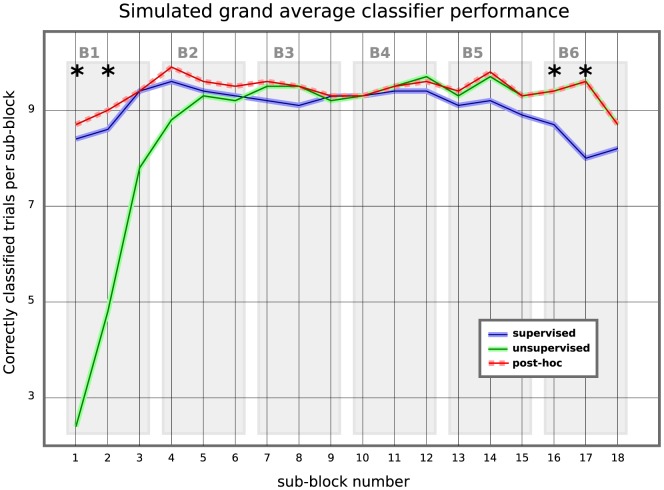
Comparison of the simulated grand average performance (n = 10) of the three classification approaches over time. The horizontal axis shows 18 sub-blocks of 5 symbol selections (10 trials) each, ordered chronologically in six experimental blocks. The performance on the vertical axis displays, how many of the 10 trials have on average been classified without fault (in absolute numbers). As not all the three classification approaches were applied online in each of the six blocks, this plot was generated by a simulated online use of the fixed supervised classifier (solid blue) and the constantly adapting unsupervised classifier (solid green) after a single initial random initialization. The unsupervised classifier was allowed to learn throughout the 18 sub-blocks without being re-set. In addition, the performance of the post-hoc unsupervised classifier is plotted (red, dotted). It has re-classified all trials in retrospection, after having learned unsupervised on the whole data from all 18 sub-blocks. Statistical significant differences between the supervised and the unsupervised performance (p = 0.05) are indicated by asterisks.

As mentioned above, it is interesting to evaluate the performance during spelling sessions of an extended duration. For this purpose we emulated a long spelling session by concatenating EEG data of the three supervised and the three unsupervised blocks per subject in chronological order. This allows the unsupervised method to improve the model by using much more data compared to the true online experiment. Furthermore, in this setting, the post-hoc classifier has seen the full data of all six blocks before it re-analyzed all trials. The grand average result for the supervised, unsupervised and post-hoc methods are compared in Fig. 8. Here we see that during the first block of 30 trials the supervised method outperforms the unsupervised and post-hoc approaches. As expected, this corroborates the true online results that we reported in the previous sections. Furthermore, when analyzing the data on a sub-block basis (10 selections each) then a paired t-test (p = 0.05) indicates that the difference in performance between supervised and unsupervised is only significant for the first 2 sub-blocks i.e. 20 trials. From that point on, and for all but the final two experimental blocks, both methods perform comparably and the difference is not statistically significant. This indicates that the proposed online experiment was especially challenging for the unsupervised method.

The post-hoc approach, which is trained (without using label information) on the entire test-set, performs relatively stable over all six blocks. The supervised approach displays a more pronounced performance drop towards the end. As the two other methods manage to maintain a high level of performance, supervised 's drop can not be caused by less informative EEG signals. On the sub-block level, the observed differences between the supervised method on the one hand, and unsupervised and post-hoc on the other hand were significant for the second to last and third to last sub-blocks (p = 0.05). Of course, this finding provides support for the use of adaptive methods, of which the unsupervised approach is a more powerful variant.

**Figure 9:**
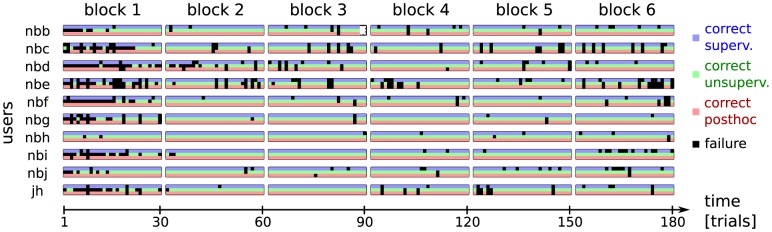
We present the individual errors made in the simulated long experiment. The unsupervised method makes most mistakes in the first block but performs at the same level as the supervised method in the following blocks. This demonstrates that the block size we used does put the unsupervised method at a disadvantage in the online study. On top of that we see that the posthoc analysis is able to correct these initial mistakes. The result is that unlike a real calibration session, the first unsupervised block can be used for communication. Finally, during the last block, the unsupervised methods perform slightly better than the supervised one.

We conclude this section by analyzing the errors on a per-subject level, like we did for the true online experiments. The individual selection errors made by the different approaches are shown in Fig. 9. In the simulation, 49% of the mistakes made by supervised were made by post-hoc, too. Furthermore, 55% of the post-hoc mistakes were committed by supervised, too. Even though we cannot make hard claims, this indicates that there might be “objectively” difficult trials.

The results from the true online experiments have revealed that unsupervised makes much more mistakes in the first thirty trials, i.e. one block. In the extended experiment we observe that for most users all three method perform similarly from the second block onwards. At this point, it is interesting to focus once more on those three blocks where unsupervised and post-hoc failed during the online sessions. This is block 1 for users *nbe* and *jh* and block 6 for user *jh*. Remarkably, in this extended experiment post-hoc makes only a few mistakes on these blocks, which indicates that the warm-up effect limited the spelling performance for unsupervised and post-hoc in the true online experiment. Furthermore, all three methods perform quite well in the problematic final block of user *nbb*. This illustrates one of the key messages of this manuscript: it is possible to train a reliable classifier without label information, but data (from 30 trials more or less) is still required to build a good decoding model.

## Discussion

This work presents an online evaluation of true zero training in a real online auditory BCI experiment with a low signal to noise ratio. Our results corroborate previous findings from the offline analysis on visual ERP data. Our online results indicate that true unsupervised spelling without any form of prior training is actually possible, but comes at a price. Compared to a typical calibration session, the unsupervised approach exhibits a warm-up period during which the user is able to interact with the system but at the cost that the output is unreliable. The length of this warm-up period is subject– and session specific. However, compared to the calibration session, the warm up period is not necessarily lost time during which no communication is possible. Instead, the classifier is able to revise and improve its initial (faulty) predictions thanks to the constant adaptation. We have shown that these revised predictions are at least as reliable as those obtained by a supervised, calibrated system. As a result, the user can effectively communicate already during the warm-up period by ignoring the mistakes and continuing to spell as if the previous selections were correct. The user knows, that the unsupervised adaptation will eventually revise these mistakes.

Nevertheless, to increase the usability of this approach, the warm-up period has to be reduced. An offline simulation on visual data, which has a higher signal to noise ratio, indicates that the warm-up period is less significant in those paradigms [1], [3]. Furthermore, it has been shown in offline analysis that the inclusion of language models is not only able to increase the reliability of the decoding, as is the case in a supervised setting [35], [51], but can also reduce the warm-up period [3]. However, the most significant reduction of the warm up period is obtained by initializing the model trough transfer learning [3]. In transfer learning, a general model is obtained using prerecorded data from different users and used as initialization for a novel user. Then during online usage, this general model is adapted to the new user. Kindermans et al. [3] have shown in a simulation of an online experiment on visual ERP data, that this approach can indeed compete with a supervised model. Even when the general model is trained without label information. We would like to stress that our novel unsupervised techniques can be readily applied also beyond EEG-based BCI. Also for invasive studies an unsupervised decoding may be highly useful to adapt for the typical nonstationarities encountered. Future studies will focus on multi-modal imaging data, where an unsupervised adaptation scheme like the one presented may enhance and speed the decoding process.
